# Intra‐Arterial Deoxyribonuclease Therapy Improves Stroke Outcomes in Aged Mice

**DOI:** 10.1111/cns.70461

**Published:** 2025-06-02

**Authors:** Junxiang Yin, Michael Wu, Jennifer White, Ellie StClair, J. Mocco, Michael F. Waters

**Affiliations:** ^1^ Department of Neurology Icahn School of Medicine at Mount Sinai New York New York USA; ^2^ Barrow‐ASU Center for Preclinical Imaging Barrow Neurological Institute Phoenix Arizona USA; ^3^ Colorado School of Mines Golden Colorado USA; ^4^ Department of Neurosurgery Icahn School of Medicine at Mount Sinai New York New York USA

**Keywords:** acute ischemic stroke, deoxyribonuclease, futile recanalization, intra‐arterial therapy, neutrophil, neutrophil extracellular traps

## Abstract

**Background:**

Futile recanalization affects more than half of acute ischemic stroke (AIS) patients. Neutrophil extracellular traps (NETs) are a major factor of microvascular hypoperfusion after stroke. Deoxyribonuclease I (DNase) targeting NETs exhibited a neuroprotective effect in young mice with AIS. This study explored a novel direct intra‐arterial administration of DNase therapy and its effect in aged mice with AIS.

**Method:**

AIS was induced in aged C57BL/6 mice followed by reperfusion and immediate, intra‐arterial DNase administration via the internal carotid artery. Cerebral blood flow (CBF), neurological function, cerebral infarct volume, and NET markers were examined.

**Results:**

Direct intra‐arterial DNase therapy significantly increased CBF, reduced neurological deficit scores, increased the latency to fall in the wire hang test, reduced cerebral infarct volume, and decreased neutrophil and NET count in both the parenchyma and micro vessels in aged mice with AIS compared with age‐matched vehicle controls.

**Conclusion:**

Our data is the first to demonstrate that successful, direct intra‐arterial DNase therapy provides more efficient cerebral reperfusion and better outcomes after recanalization during the treatment of large vessel occlusion in aged mice. This study provides evidence for the potential clinical application of catheter‐delivered intra‐arterial DNase therapy post‐recanalization.

## Graphical abstract

We explored a novel direct intra‐arterial DNase therapy and its effect in aged mice with AIS. IA DNase therapy decreased neutrophil and NET burden in the penumbra in aged mice with AIS, provided more efficient cerebral reperfusion, and better functional outcomes after recanalization during the treatment of stroke.

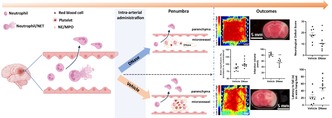

## 1 Introduction

Stroke is the third‐leading cause of mortality and disability in the world [1, 2]. More than 30% of acute ischemic strokes (AIS) are large vessel occlusion (LVO), disproportionately contributing to 95% of mortalities and 62% of long‐term disability in stroke patients [3]. Endovascular thrombectomy (EVT) is the standard of care for LVOs [4, 5, 6]. Despite there being high success rates of recanalization with EVT, more than half of patients still suffer poor outcomes and low micro‐perfusion, a phenomenon coined futile recanalization (FR) [7, 8, 9, 10]. This FR is recognized as a challenge during AIS treatment, and teasing out its mechanisms is integral to improving the outcomes of stroke patients [11, 12, 13]. Numerous factors are associated with the failure to restore penumbral microcirculation, one of which is microvascular thrombosis resulting in distal capillary hypoperfusion.

Neutrophil Extracellular Traps (NETs) are extracellular deoxyribonucleic acid (DNA) networks wrapped around histones and granular proteins produced and extruded by activated neutrophils. Neutrophils and NETs are important components of cerebral thrombi [14] and markers of NET formation are also significantly increased in the plasma and cerebral penumbra of ischemic stroke patients [15, 16]. Both neutrophils and NETs are elevated in the central nervous system and in the peripheral circulation of ischemic stroke patients and animal models of ischemic stroke. Elevated plasma NET biomarkers are also correlated with worse stroke outcomes [15, 16, 17, 18]. Additionally, NETs have been identified as major triggers and structural factors of microvascular thrombosis, and they exacerbate microvascular hypoperfusion in stroke [16, 19, 20]. Furthermore, NETs are thought to play a key mechanistic role in thrombolysis resistance [21]. Recent studies have also reported that an early decline in deoxyribonuclease (DNase) activity and elevated NET marker concentrations in circulation increased the risk of delayed cerebral ischemia following hemorrhagic stroke [22, 23]. Importantly, older age is associated with increased NET formation in the ischemic brain, contributing to increased risk and worse outcomes from stroke [24]. By cleaving DNA, DNase has been proposed as an efficient antithrombotic drug [25]. Additionally, DNase has exhibited promising neuroprotective effects in AIS via targeting NETs in young ischemic stroke mice [14, 17, 18]. Intraperitoneal injection (IP) and intravenous tail vein injection (IV) are two of the most common administration routes in small animal experiments. However, IP injection is unlikely to be clinically translatable. Tail vein injection is a systemic administration requiring a high dose, and potential repeated administrations are limited via tail vein injection. To date, there are no FDA‐approved therapies for modifying NETs in stroke. To explore a more efficient, clinically translatable, and low‐dose strategy for DNase therapy and its effects during the treatment of AIS, we investigated a novel, direct intra‐arterial DNase therapy in aged mice with AIS.

## 2 Materials and Methods

### 2.1 Animals

All wild type C57BL/6 mice were purchased from the Jackson Laboratory (Bar Harbor, ME) for this study. 6‐month‐old wild type C57BL/6 male mice were used in the DNase activity test and methylene blue intra‐arterial injection. There was a total of 40 aged C57BL/6 mice (~20 months) used in DNase/Vehicle treatment after AIS. The first cohort of aged mice (10 female mice and 10 male mice) was used in IP DNase/Vehicle treatment to evaluate the effect of DNase on vascular density after stroke. The second cohort of aged mice (10 female mice and 10 male mice) was used in intra‐arterial DNase/Vehicle treatment. All experiments involving animals were performed following the protocols in accordance with the Revised Guide for the Care and Use of Laboratory Animals and were approved by the Institutional Animal Care and Use Committee (IACUC) of the Icahn School of Medicine at Mount Sinai. The study was carried out in compliance with the ARRIVE guidelines. All methods were performed in accordance with the relevant guidelines and regulations.

### 2.2 Deoxyribonuclease I (DNase) Activity Assessment In Vitro and In Vivo

DNase activity in vitro test: Recombinant DNase I (DNase, Sigma‐#4536282001) was diluted with pure water into different concentrations (0.01, 0.001, 0.0001, 0.00001 μg/μl); heat‐inactivated DNase was used as a control (HI‐DNase, 0.01 μg/μl). DNase activity was measured with a DNase activity fluorescent assay kit (Acrobiosystems, #ASE‐1002). Relative fluorescence units (RFU) were used for DNase activity curve analysis. DNase activity in vivo test: 6‐month‐old wild type C57BL/6 male mice received DNase (6 mg/kg, 1 Unit DNase = 1 ug DNase) via intraperitoneal (IP) injection (*n* = 3) or tail vein (IV) injection (*n* = 4). Age‐matched wild type mice receiving a vehicle injection were used as controls (Ctrl, *n* = 3). Serum was collected 30 min after DNase or vehicle administration and used to evaluate DNase activity.

### 2.3 Transient Middle Cerebral Artery Occlusion (MCAO)

Mice were subjected to the intraluminal filament method of transient middle cerebral artery occlusion (MCAO) as described previously [26]. Briefly, mice were anesthetized and placed in the supine position on a heating pad, and body temperature was monitored using the small animal anesthesia system (Kent Scientific Cor. FL). Under a stereo dissecting microscope (AmScope, CA), the right common carotid artery (CCA), external carotid artery (ECA), and internal carotid artery (ICA) were exposed and isolated. To induce MCAO, a silicon‐coated nylon selected based on mice body weight (Doccol, MA) was introduced into the internal carotid artery through the external carotid artery, then further advanced to the origin of the MCA. The monofilament for each mouse was selected based on the body weight of each mouse according to the manufacturer's recommendation. Doccol filament #602323PK10 (diameter 0.23 mm, coating length 2‐3 mm) was used for mice with a body weight of 30 ± 5 g, and Doccol filament #602423PK10 (diameter 0.24 mm, coating length 2‐3 mm) was used for mice with a body weight of 40 ± 5 g. The 2D laser speckle system (PeriCam PSI‐Z, Perimed) was used to automatically quantify and calculate the average signal intensity of cerebral blood flow (CBF) for a defined region of interest (ROI), a 3‐mm diameter area that locates the region supplied by the middle cerebral artery. The CBF percentage at ischemia and reperfusion was used to clarify whether successful ischemia–reperfusion injury was achieved. Successful ischemia was defined as an equal or more than 75% decrease in CBF based on the relative percentage of the intensity of CBF collected from the ROIs of ipsilateral vs. contralateral according to the image scanned with 2D laser images. After 60 min of ischemia, the filament was fully withdrawn for reperfusion. Successful reperfusion is defined as a 75% restoration of the relative percentage of the intensity of CBF. Buprenorphine SR was administered at 0.05 mg/kg of body weight to alleviate any postoperative pain, and the mice were put into a portable animal intensive care unit to recover before being transferred to their home cages.

### 2.4 Evaluation of Cerebral Blood Flow

CBF was evaluated using a 2D laser speckle system (PeriCam PSI‐Z, Perimed). Mice were anesthetized and laid prone on a pre‐warmed SurgiSuite surgical platform (Kent Scientific, CT). A small incision was made, and the skin was retracted to expose the intact skull. CBF from all mice was collected and evaluated at four time points: baseline (before surgery), ischemia (during occlusion and ischemia), reperfusion (after filament withdrawn), and treatment (immediately after compound was intra‐arterially administered). The degree of restoration of the CBF after treatment is the indicator of the efficacy of the treatment. Average signal intensity of ROIs was analyzed from both ipsilateral and contralateral sides. The analysis data of CBF was reported as a percentage of ipsilateral CBF vs. contralateral CBF to indicate reperfusion efficacy. Evaluations were performed blinded to the treatment group (DNase or vehicle).

### 2.5 Intra‐Arterial (IA) Injection Procedure

To generate a successful IA injection procedure, microcatheters (Doccol, MA) and Hamilton microliter syringe (Hamilton, NV,100 μL) were used for injection. Mice were subjected to MCAO. At the beginning of reperfusion, the microcatheter was inserted carefully into the internal carotid artery via the same surgical path used for occluding the MCA. The microcatheter was secured in place, and the plunger on the Hamilton syringe was deployed so that 20 μL of methylene blue was incrementally administered over the course of 5 min. After administration, the microcatheter was kept in place for 2 min to mitigate backflow before being withdrawn, and the surgical site was closed as described above. Mice were sacrificed within 10 min after the microcatheter was withdrawn, and brains were extracted without prior transcardial perfusion. 2 mm serial sections were cut to display the distribution of dye in the cerebral vessels and parenchyma.

#### 2.5.1 IA DNase Administration

Mice were randomly assigned to receive either the IA DNase or Vehicle treatment. The microcatheter was introduced to the internal carotid artery as described above. 25 μg/25 μL DNase (*n* = 10) or 25 μL normal saline (vehicle, *n* = 10) was administered as described above. After administration, the microcatheter was withdrawn, the external carotid artery was permanently ligated, and the ICA and CCA were reopened. Post‐operative care was performed as described in the above survival surgery. CBF was re‐evaluated and recorded before mice were placed in the intensive care unit for recovery.

### 2.6 Intraperitoneal (IP) DNase Treatment

IP DNase administration: Mice were administered 50 μg/100 μL deoxyribonuclease I (DNase) (*n* = 10) or normal saline (vehicle, *n* = 10) via IP injection immediately after establishing reperfusion.

### 2.7 Point Sensorimotor Neurological Deficit Score (NDS)

At 24 h post‐stroke, a 28‐point rodent neurological deficit score (NDS) was used to evaluate sensorimotor function as described previously [27, 28]. The NDS uses a 0 (no deficits) to 4 (extreme deficits) point system on 7 sensorimotor functions: body symmetry, gait (open bench top), circling behavior, assessment of climbing, assessment of front limb symmetry, compulsory circling, and whisker response. To reduce bias in data collection, all mice, experimental groups, and treatments were randomly assigned and coded such that outcome investigators were blinded. All behavior tests were scored independently by two investigators who were blinded to the identity of the individual animals, and their scores were averaged.

### 2.8 Wire Hang Test

The wire hang test assesses locomotor ability [29]. At 24 h post‐stroke, mice were brought to the wire and allowed to grasp the wire only with their two forepaws. The mice were released and allowed to hang for a maximum of 2 min. Latency to fall (sec) was recorded to evaluate grip strength and endurance.

### 2.9 Triphenyltetrazolium Chloride (TTC) Staining

At 24 h post‐stroke, mice were sacrificed and transcardially perfused with 0.01 M phosphate buffered saline (PBS) solution (pH 7.6). Whole brains were extracted and chilled on dry ice. Partially frozen brains were then sectioned into 2 mm coronal slices and incubated in 1.5% prewarmed 2,3,5‐Triphenyltetrazolium chloride (TTC) solution. Slices were incubated until clear infarct boundaries were observed on both sides of each section. Sections were transferred into a 4% paraformaldehyde solution at 4°C overnight and scanned the next day using an HP Scanjet 5590. Postfixed brain slices were cryoprotected with 30% sucrose in PBS overnight in preparation for serial coronal cryostat sections (16 μm).

### 2.10 Assessment of Ischemic Infarct Volume

The infarct volume was calculated blindly based on scanned images of TTC‐stained coronal sections as previously described [30, 31]. Briefly, infarct borders were drawn on each scan using National Institute of Health ImageJ 1.54f, and computer analysis was used to calculate the infarct area of the anterior and posterior surfaces for each coronal section. Infarct area was corrected for edema by determining the ratio of the area of the contralateral hemisphere to that of the ipsilateral hemisphere. This ratio was then multiplied by the unadjusted infarct area to obtain the final edema‐adjusted infarct areas. Edema‐adjusted infarct volume for each section was then estimated by taking the average between the adjusted posterior and anterior infarct areas for each section and multiplying it by the slice thickness (2 mm). Total adjusted infarct volume was obtained by finding the sum of the adjusted infarct volumes of all coronal sections for each brain.

### 2.11 Immunohistochemistry Staining, Immunofluorescence Staining, and Imaging

Cryostat sections were thawed, washed, and rehydrated. For immunohistochemistry (IHC) staining, sections were blocked with 3% animal serum in PBS‐T for 1 h and then incubated with anti‐Ly6G (BD Bioscience, #551459) primary antibody overnight at 4°C. Sections were washed and incubated in anti‐rabbit Horseradish peroxidase secondary antibody. Sections were then treated with DAB substrate working solution (Vector Laboratories, #SK‐4100). Hematoxylin was used to counterstain cell nuclei. Images were collected using a Zeiss microscope and post‐processed using ImageJ. For immunofluorescence (IF) staining, the sections were washed and rehydrated. Sections were incubated with the following primary antibodies: anti‐Ly6G (BD Bioscience, #551459), anti‐Citrullinated Histone H3 (CitH3, Cell Signaling, #97272), anti‐Neutrophil Elastase conjugated with Alexa Fluor 555 (NE, Cell Signaling, #39680) anti‐Vascular cell adhesion molecule 1 (VCAM1, Thermo Fisher, #MA5‐11447), anti‐myeloperoxidase (MPO, Bioss, BS‐4943R), or Lectin—Dylight 488 (Vector, #DL‐1174‐1) overnight at 4°C. A secondary antibody (Alexa 488 or Alexa 568, Thermo Fisher) incubation was performed if necessary, and DAPI counterstained cell nuclei. Images were collected using a Nikon confocal microscope and post‐processed using Nikon Microscope Imaging Software and ImageJ. For cell counts, three 200 × 200 pixel regions of interest (ROIs) were drawn and placed over three representative areas within a 10× image taken from the brain sections of each animal. Cells were hand counted within each ROI, and the average between all three ROIs was calculated for further analysis. Cell counts were collected using ImageJ and analyzed in GraphPad Prism.

### 2.12 Capillaries Density

Mice were subjected to MCAO as previously described. After reperfusion, mice were administered either 50 μg/100 μl DNase or normal saline solution through IP injection. To examine the capillary density, we used a methodology that originally combined blood vessel lumen staining with CLARITY [32, 33, 34]. A brief description is as follows: Mice were sacrificed at 24 h after reperfusion and transcardially perfused with PBS followed by 4% paraformaldehyde in PBS. This was followed by perfusion with 10 mL of 2% gelatin combined with albumin‐FITC (Sigma, 0.125% w/v). The brain was extracted and incubated in 4% PFA overnight at 4°C. Postfixed brains were incubated in 15%, then 30% sucrose (pH 7.4). Serial coronal sections (16 μm) were prepared and stained with anti‐NeuN antibody (Cell Signaling, #24307) with secondary antibody Alexa 555 (Thermo Fisher, # A‐21428) and sections were imaged on a Zeiss confocal laser microscope (LSM700, Carl Zeiss Microscopy). Vascular density in the penumbra was evaluated semi‐quantitatively by comparing relative fluorescent (Albumin‐FITC) units per ROI between the treatment and vehicle control groups.

### 2.13 Quantification of NETs Biomarkers Concentration in Plasma

Whole blood was centrifuged at 1500 g for 15 min at 4°C for plasma collection. Plasma levels of cell‐free DNA were measured by Quant‐iT PicoGreen dsDNA Assay kit (ThermoFisher, #P11496) following the manufacturer's protocol. Enzyme‐linked immunosorbent assay (ELISA) kits were used to determine the plasma levels of citrullinated histone H3 (CitH3 (Clone 11D3) ELISA Kit, Cayman Chemicals, #501620), myeloperoxidase (Mouse MPO Quantikine ELISA kit, ThermoFisher, #EMMPO), and neutrophil elastase (Mouse Elastase Platinum ELISA, Abcam, #ab252356) according to each respective manufacturer's protocol.

### 2.14 Statistical Analysis

All data are presented as mean ± standard error of mean. All data sets follow a normal distribution as determined by the Shapiro–Wilk test. An unpaired *t*‐test was used for data analysis from two groups, and one‐way ANOVA with post hoc Tukey's test was applied for the data from multiple groups. GraphPad Prism 10 (GraphPad, MA) was used for statistical analysis, DNase activity curve, and graphs generation. Statistical significance was defined as *p* < 0.05 for all analyses.

## 3 Results

### 3.1 Evaluation of Deoxyribonuclease I (DNase) Activity In Vitro and In Vivo

**FIGURE 1:**
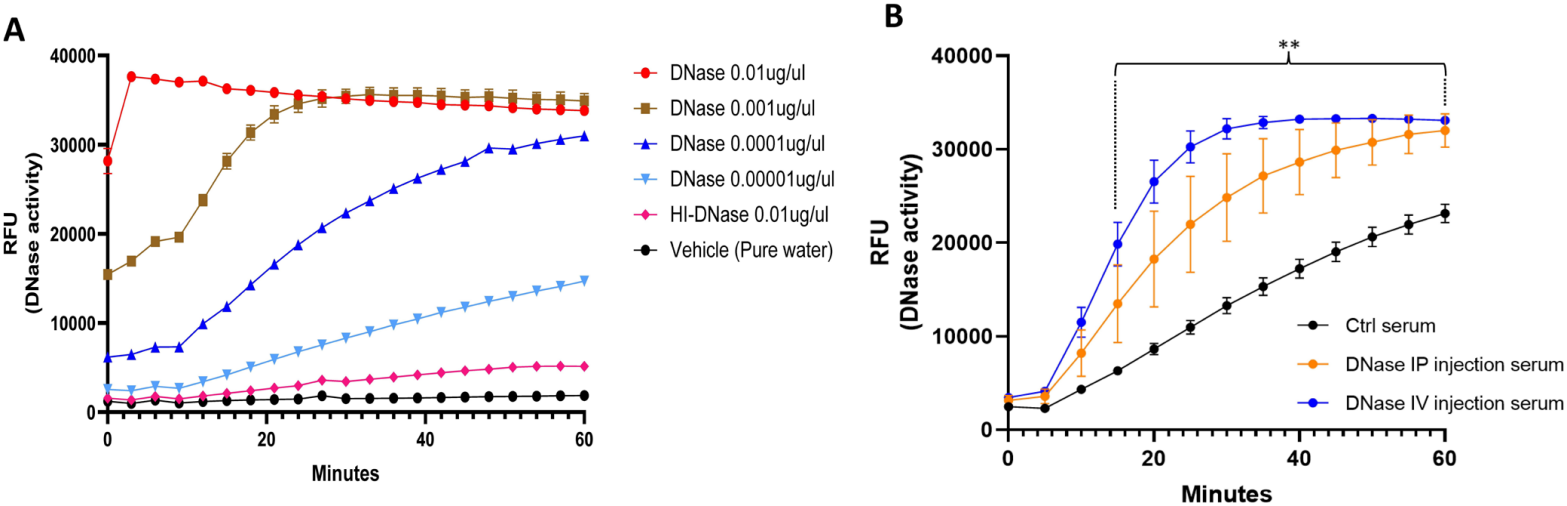
DNase activity in vitro and in vivo. (A) Dose dependent DNase activity curve in vitro. (B) DNase activity curve in mice serum. DNase IP injection, *n* = 3; tail vein (IV) injection, *n* = 4, and Ctrl, *n* = 3. **p < 0.01, both IP and TV injection serum vs. Ctrl serum. GraphPad Prism 9.5 was used for data and analysis and DNase activity curve generation.

To confirm the activity of Deoxyribonuclease I (DNase), our in vitro study showed that DNase exhibited a reliable dose‐dependent activity curve (Figure 1A). Our in vivo experiment detected the activity curve of serum DNase in mice 30 min after IV or IP DNase administration (Figure 1B). The activity curve showed serum DNase activity was significantly elevated in both IP and IV groups in comparison to control serum. However, DNase via IV injection generated a quicker and stronger activity curve compared with DNase via IP injection. Endogenous DNase activity in vehicle controls only exhibited a linear activity curve (Figure 1B). This data indicate that DNase supplementation improves in vivo DNase activity, and that the method of administration has a significant effect on early DNase activity.

### 3.2 Procedure of Successful Intra‐Arterial (IA) Injection in Mice With AIS

**FIGURE 2:**
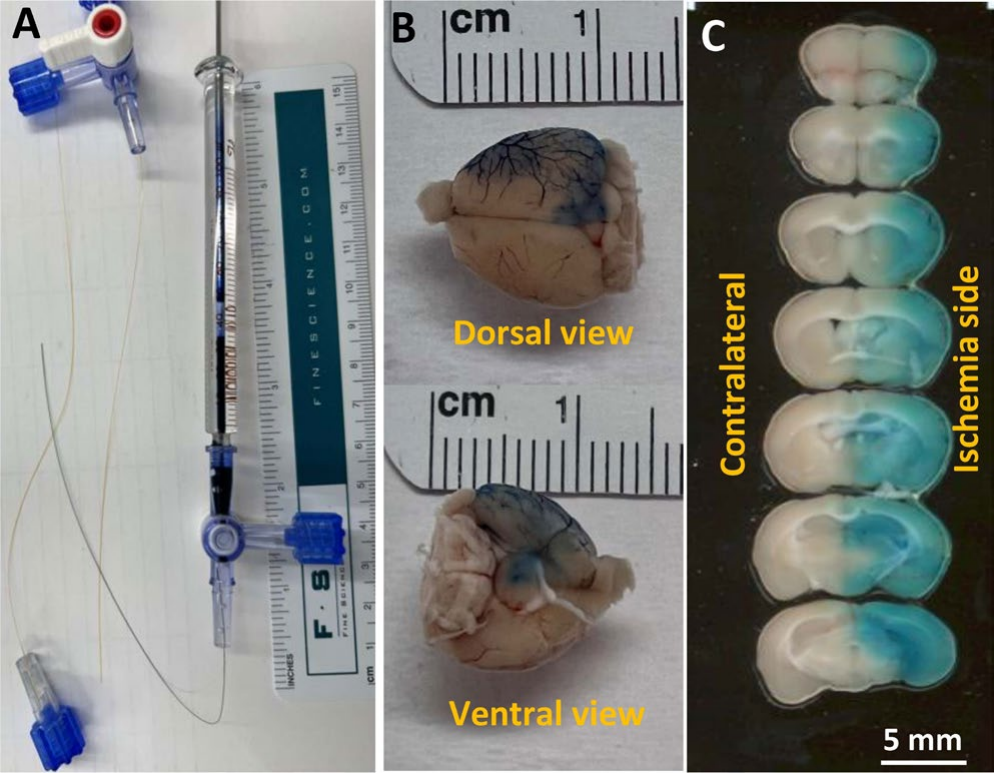
Intra‐artery injection tools and Methylene blue injection after MCAO. (A) Microcatheters and Hamilton syringe. (B) Images of mouse brain after direct intra‐artery Methylene blue injection, mouse was subjected to MCAO with 60′ ischemia followed by reperfusion and immediate intra‐arterial 20 μL Methylene blue injection. (C) Images of brain sections. With immediate intra‐arterial methylene blue injection, blue dye spreads in ischemic reperfusion brain tissue side and no blue dye were observed in contralateral brain tissue.

In this study, we focused on modeling a protocol that would have meaningful and immediate translation to current clinical practice for stroke therapy. Thus, we explored IA injection as a means of directly administering low‐dose DNase to a local cerebral area. To build a successful IA injection procedure, microcatheters attached to a Hamilton microliter syringe were used to inject. We first utilized methylene blue to gauge distribution via this method and as a proof‐of‐concept demonstration. Methylene blue was directly administered intra‐arterially after MCAO and recanalization (Figure 2A). Brain surface imaging clearly showed that the methylene blue was distributed along the micro vessels supplied by the middle cerebral artery in the dorsal and ventral views of the right hemisphere of the mouse brain (Figure 2B). Coronal sections revealed that methylene blue was disseminated throughout the ischemic, but not contralateral hemisphere (Figure 2C). Methylene blue was localized to the vasculature and parenchyma of the ischemic hemisphere but not the contralateral hemisphere. This indicates that methylene blue was successfully delivered via direct IA injection into the ischemic area of the mouse brain and suggests that IA administration can successfully deliver local, low‐dose therapeutics for LVOs.

### 3.3 IA DNase Treatment Improved Neurological Function and Reduced Infarct Volume via Increasing Efficient Cerebral Reperfusion in Aged Mice With AIS

**FIGURE 3:**
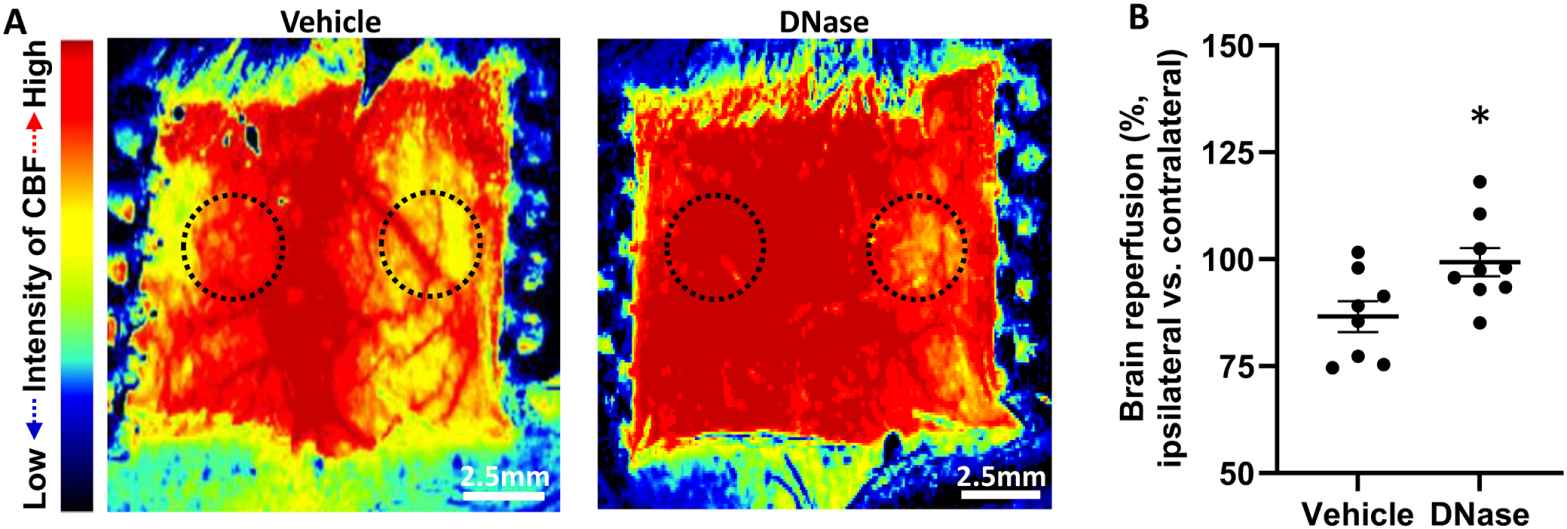
Intra‐arterial DNase treatment improved early cerebral blood reperfusion in aged mice with AIS. Cerebral blood flow was evaluated immediately after intra‐arterial DNase injection. The ratio of cerebral blood flow (ipsilateral (ischemic) side to contralateral side) was calculated. (A) Representatives of cerebral blood flow images for Vehicle and DNase, and (B) Results of the ratio of cerebral blood flow. *p < 0.05, compared with Vehicle.

NETs contribute towards microcirculation hypoperfusion and are associated with worse prognosis [15, 16, 17, 19, 20]. Here, we are the first to investigate the effect of IA DNase administration in aged mice with AIS. In our study, laser imaging revealed that IA DNase injections post AIS improved reperfusion. The signal intensity ratios of CBF images showed that IA DNase administration significantly improved CBF post‐reperfusion in comparison to vehicle (Figure 3A,B). This emphasizes the potential of IA DNase administration in improving reperfusion efficiency in microvascular capillary beds post mechanical thrombectomy. All images of CBF from different time points were presented in Figure S1.

**FIGURE 4:**
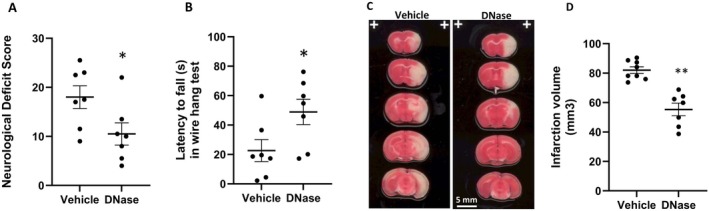
Intra‐arterial DNase treatment improved neurological function outcomes and reduced ischemic infarction in aged mice with AIS. (A) Neurological deficit score, (B) Latency to fall in wire hang test, (C) Representative TTC images, (D) Bar graph of infarct volumes. ImageJ was used for infarct volume calculation based on TTC staining images. *p < 0.05, **p < 0.01, compared with vehicle.

This improved reperfusion efficiency corresponds with an improvement in functional neurological outcomes and a reduction in ischemic infarct volume. IA DNase administration significantly reduced NDSs and increased the latency to fall in the wire hang test in comparison to those of mice receiving vehicle treatment (Figure 4A,B). These findings suggest that IA administration of DNase post‐reperfusion improves sensorimotor outcomes in AIS mice. Additionally, IA DNase administration post‐reperfusion produced an obvious reduction in stroke volume in comparison to vehicle controls (Figure 4C). Volume quantifications confirmed a significant reduction in infarct volume in the IA DNase group in comparison to that of the vehicle controls (Figure 4D). Taken together, these findings point to IA DNase administration post‐recanalization improving stroke outcomes in AIS mice, possibly by mitigating futile reperfusion and improving CBF. All detailed analyzed images and data were presented in Figure S2, Figure 3, and Data S1.

### 3.4 IA DNase Reduced NETs Formation in the Penumbra of Aged Mice With AIS

**FIGURE 5:**
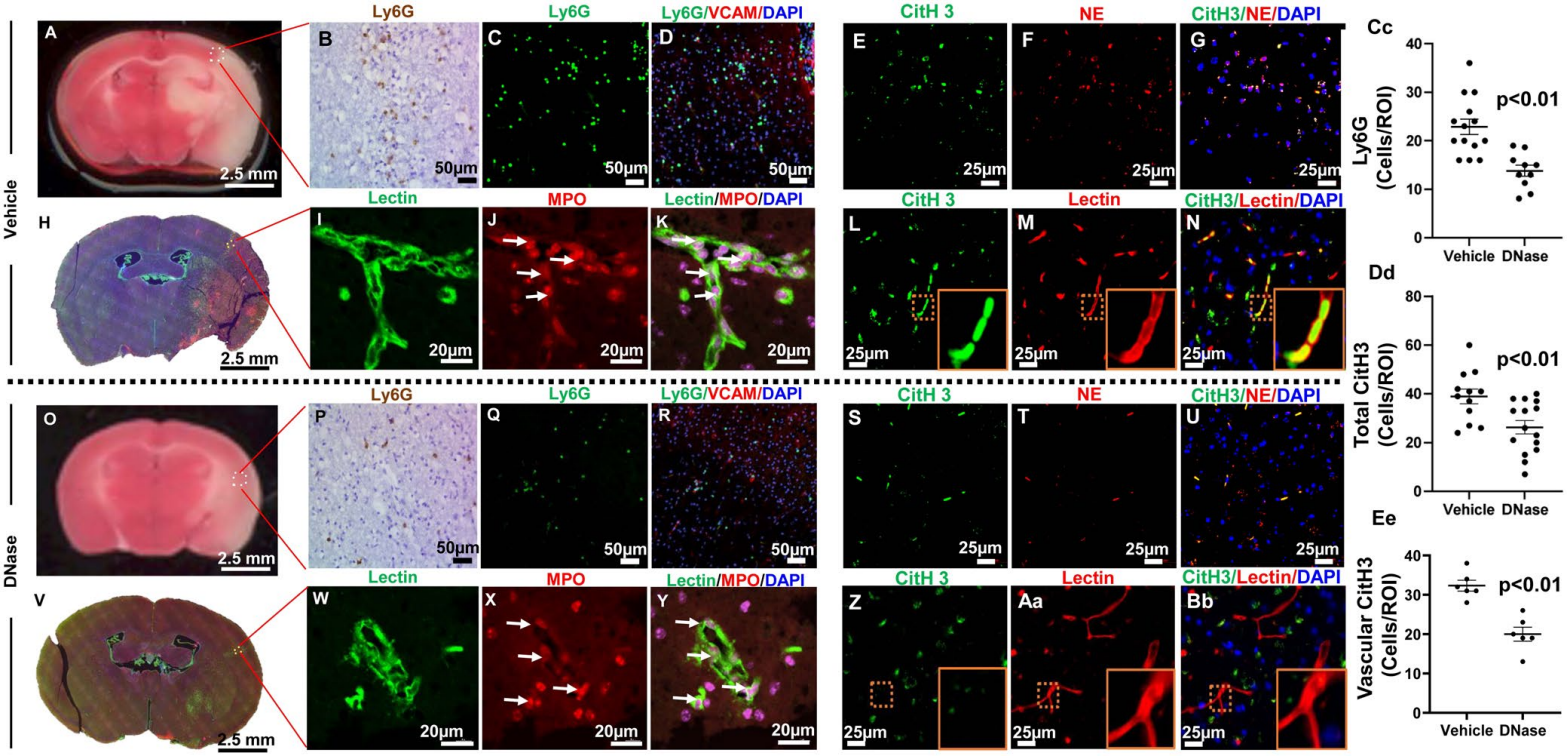
Intra‐arterial DNase treatment reduced neutrophils infiltration and NETs formation in ischemic penumbra of aged mice with AIS. Brain sections were stained with neutrophil specific markers (Ly6G, myeloperoxidase‐MPO, and neutrophil elastase‐NE), NETs marker (Citrullinated histone H3‐CitH3), vascular marker antibodies (vascular cell adhesion molecule 1 [VCAM] and Lectin), and nuclei (DAPI). NETs were identified by neutrophil specific markers and CitH3 positive signaling. Representative images from the vehicle treatment group: (A) Image of TTC staining, penumbra area recircled with white dash line. (B) Image of anti‐Ly6G IHC staining. (C) Image of anti‐Ly6G IF staining. (D) Merge image of anti‐Ly6G (green), anti‐VCAM (red), and DAPI (blue) IF staining. (E) Image of anti‐CitH3 IF staining. (F) Image of anti‐NE IF staining. (G) Merge image of anti‐CitH3 (green), anti‐NE (red), and DAPI (blue) IF staining. (H) Merge image of anti‐Lectin (green), anti‐MPO (red) and DAPI (blue) of whole section IF staining. (I) Image of anti‐Lectin IF staining. (J) Image of anti‐MPO IF staining, intra‐vascular (white arrow). (K) Merged image of anti‐Lectin, anti‐MPO and DAPI, intra‐vascular NETs signal (white arrow) in penumbra. (L) Image of anti‐CitH3 IF staining. (M) Image of anti‐Lectin IF staining. (N) Merged image of anti‐CitH3 (green), anti‐Lectin (red) and DAPI (blue), CitH3 signal (yellow color) in vascular (right low corner panel) in penumbra. Representative images from the DNase treatment group: (O) Image of TTC staining, penumbra area recircled with white dash line. (P) Image of anti‐Ly6G IHC staining. (Q) Image of anti‐Ly6G IF staining. (R) Merged image of anti‐Ly6G (green), anti‐VCAM (red) and DAPI (blue) IF staining. (S) Image of anti‐CitH3 IF staining. (T) Image of anti‐NE IF staining. (U) Merge image of anti‐CitH3 (green), anti‐NE (red) and DAPI (blue) IF staining. (W) Image of anti‐Lectin IF staining. (V) Merge image of anti‐Lectin (green), anti‐MPO (red) and DAPI (blue) of whole section IF staining. (X) Image of anti‐MPO IF staining, intra‐vascular (white arrow). (Y) Merged image of anti‐Lectin, anti‐MPO, and DAPI IF staining, intra‐vascular NETs signal (white arrow) in penumbra. (Z) Image of anti‐CitH3 IF staining. (Aa) Image of anti‐Lectin IF staining. (Bb) Merge image of anti‐CitH3 (green), anti‐Lectin (red), and DAPI (blue) IF staining. (Cc) Plots graph of the number of anti‐Ly6G positive signal. (Dd) Plots graph of the number of total CitH3 positive signal. (Ee) Plots graph of the number of vascular CitH3 positive signal. **p < 0.01, compared with Vehicle.

To confirm the effect of IA DNase administration on NET formation, we evaluated neutrophil infiltration and NET formation in both the parenchyma and micro vessels in the penumbra of mice with AIS using IHC and IF staining. Fixed‐frozen brain tissue was sectioned and stained with neutrophil markers (Ly6G, MPO, and NE) and NET markers (CitH3), as well as vascular markers (VCAM and Lectin) (Figure 5). The images show abundant strong neutrophil and CitH3 signals in the parenchyma and micro vessels in the penumbra of mice from the vehicle treatment group (Figure 5B–G,I–N). Conversely, DNase reduced the number of neutrophils and CitH3 significantly in the parenchyma and micro vessels in the penumbra of AIS mice (Figure 5P–U,W–Bb). Statistical analysis indicated that DNase treatment significantly reduced the infiltrated neutrophils count and CitH3 signal in parenchyma and vessels compared to vehicle treatment (Figure 5Cc–Ee). These findings indicate that IA DNase administration reduces both the number of neutrophils and NETs in the penumbra of AIS mice in comparison to vehicle controls.

### 3.5 IP DNase Treatment Reduced NETs Formation in Systemic Circulation and Improved Vascular Recanalization in the Penumbra of Mice With AIS

**FIGURE 6:**
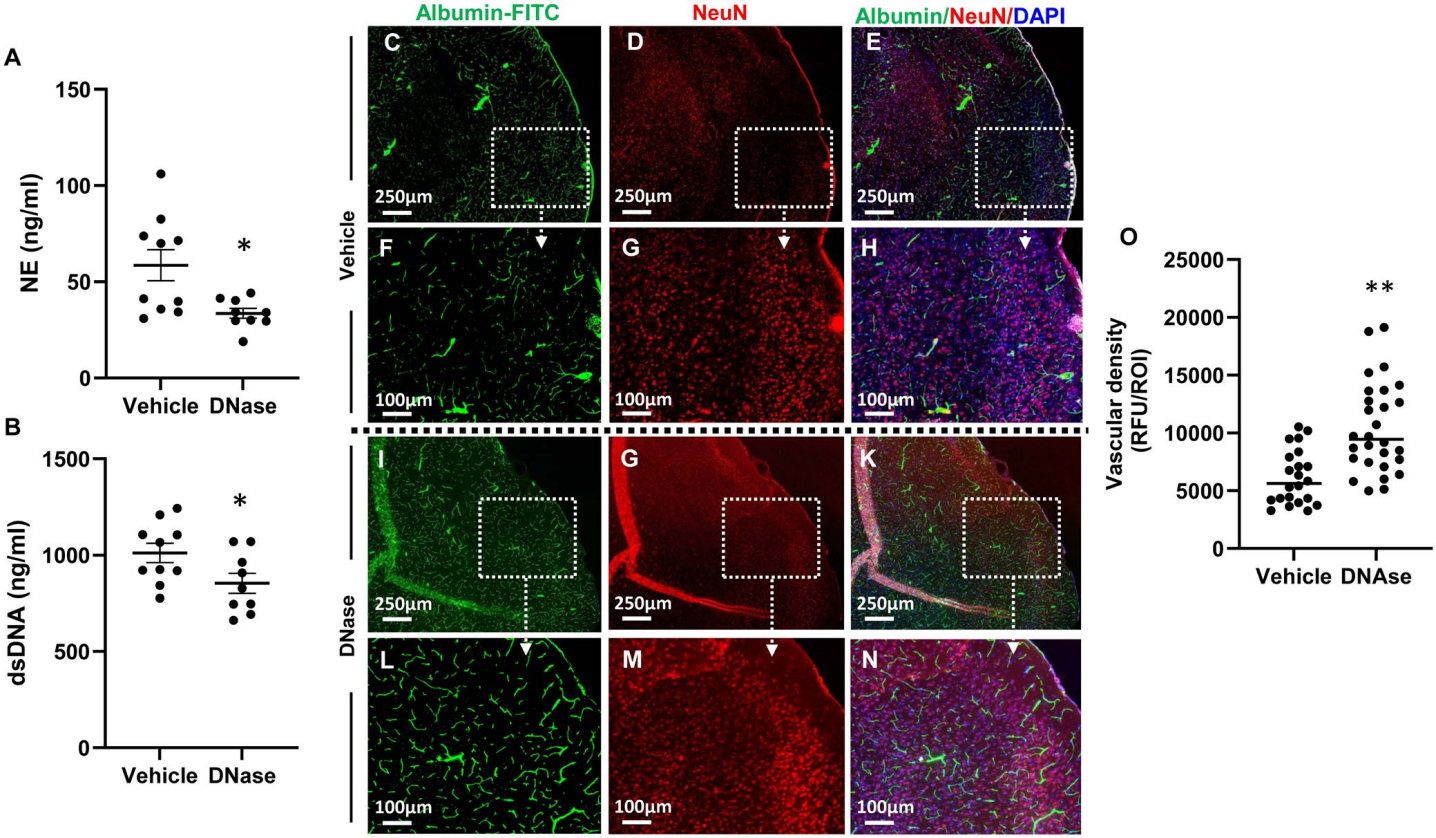
Intraperitoneal DNase treatment reduced NETs formation in systemic circulation and improved capillary recanalization in penumbra of mice with AIS. (A) Plasma NE concentration. (B) Plasma dsDNA concentration. Representative images from vehicle treatment group: (C, F) Image of Albumin‐FITC. (D, G) Image of anti‐NeuN IF staining. (E, H) Merge image of Albumin‐FITC (green), anti‐NeuN (red), and DAPI (blue) IF staining. Representative images from DNase treatment group: (I, L) Image of Albumin‐FITC. (J, M) Image of anti‐NeuN IF staining. (K, N) Merge image of Albumin‐FITC (green), anti‐NeuN (red), and DAPI (blue) IF staining. (O) Vascular density as calculated by Albumin‐FITC fluorescent signal (vehicle treatment, *n* = 6 and DNase treatment, *n* = 9). *p < 0.05, **p < 0.01, compared with vehicle.

DNase treatment not only reduced NET burden in the ischemic penumbra, but also in systemic circulation. Our data showed that IP DNase treatment significantly reduced the plasma concentration of NE and dsDNA in mice with AIS when compared to that of the vehicle treatment group (Figure 6A,B). NET‐containing cerebral microvascular thrombi may occlude capillary blood flow and induce penumbral hypoperfusion in stroke [14, 16, 19, 20]. To evaluate whether DNase treatment also improves circulation after recanalization, we tested the density of vessels in the ischemic penumbra of AIS‐aged mice. Our data showed that the vascular density in the penumbra of DNase‐treated, aged mice with AIS was significantly higher than that of vehicle‐treated, aged mice with AIS (Figure 6C,D). This data suggests that DNase improves perfusion in the ischemic penumbra, potentially by degrading intravascular NETs, particularly in microvessels. Taken together, these findings indicate that DNase treatment reduces NET burden in vivo and provides a mechanistic explanation as to how DNase improves reperfusion and stroke outcomes in AIS.

## 4 Discussion

Neutrophils are the first cell type to infiltrate ischemic tissue. Once there, they release web‐like DNA structures (NETs) in the diseased brain parenchyma and cerebral microvasculature [35, 36, 37, 38]. NET formation facilitates thrombosis and contributes to reperfusion resistance during the thrombolytic treatment of AIS [19, 39]. Additionally, plasma NET markers are significantly increased in ischemic stroke patients, are correlated with worse outcomes, and are used to predict stroke mortality and morbidity [15, 16, 17, 18, 39, 40, 41, 42]. Thus, approaches to reduce NETs have pro‐thrombolytic potential in the treatment of AIS [14, 43, 44, 45, 46, 47, 48].

DNase is FDA‐approved for the treatment of cystic fibrosis [49, 50, 51] and its safety and clinical efficacy in treating respiratory dysfunction is well established [51, 52]. However, there are no FDA‐approved therapies for modifying NETs with respect to stroke. Intravascularly, DNase breaks down NETs and other cell‐free DNA, reducing blood viscosity and resolving vascular congestion [51]. Both IP and tail vein injection of DNase therapy exhibited promising neuroprotective effects and improved neurological outcomes in young mice when given before and after AIS, suggesting the targeting of NETs with DNase as a promising therapeutic in AIS [14, 17, 19, 53, 54].

Our study of in vitro and in vivo DNase activity describes the comparative efficacy of tail vein versus IP, which was previously unresolved. Not surprisingly, we found that the activity of DNase via tail vein administration was stronger and faster than that administered through IP. Importantly, this finding suggested to us that direct intra‐arterial DNase administration may exhibit an even more robust response given its immediate application downstream of the occlusion site.

Therefore, we investigated direct intra‐arterial administration as a new procedure in the delivery of stroke therapeutics and examined the effects of IA DNase administration in aged mice of both sexes with AIS. Similar to our own findings, a recent study reported that reperfusion was significantly impaired in aged mice following recanalization of MCA occlusion [24]. Moreover, they reported vascular occlusions in the ischemic penumbra were associated with increased neutrophil infiltration and NETs. These phenomena were worse in aged mice, suggesting that the effects of mitigating NET pathology could have a greater benefit in aged subjects with AIS. Our direct intra‐arterial DNase treatment increased CBF and improved stroke outcomes in aged mice with AIS (Figures 3 and 4). Additionally, IP DNase treatment prevented the reduction of vascular density and reduced plasma NET markers (NE and dsDNA) in aged mice with AIS (Figures 5 and 6). Taking both our and others' findings [24], it is hypothesized that the reduction of neutrophil infiltration and the disruption of microvascular NETs (Figures 5 and 6) reduces the formation of microvascular occlusions and thus mitigates FR.

FR continues to pose problems for physicians in the treatment of LVOs. Intravascular administration of tPA after mechanical thrombectomy has been proposed as a treatment for FR [55]. Despite some preliminary reports that mechanical thrombectomy with tPA improves stroke outcomes, others have shown that intravenous tPA increased the occurrence of hemorrhagic transformation and intracerebral hemorrhage in patients undergoing mechanical thrombectomy [56, 57, 58]. Interestingly, in addition to the disruption of NETs and providing neuroprotection in ischemic injury, DNase reduces tPA‐associated hemorrhagic transformation by mitigating tPA‐induced blood‐brain barrier breakdown and reducing tPA‐associated hemorrhagic transformation [18, 59]. Direct intra‐arterial DNase‐tPA coadministration at the occlusion site may similarly reduce hemorrhagic transformation seen in mechanical thrombectomy with intra‐arterial tPA treatment while maintaining its positive outcomes. Further study will be needed to test the safety of intra‐arterial tPA‐DNase coadministration after mechanical thrombectomy and whether there is a clear clinical benefit to coadministration in comparison to intra‐arterial DNase on its own.

There were several limitations in this study. Firstly, for the in vivo DNase activity test, all mice were male 6‐month‐old C57BL/6 mice. While this test is important in showing that DNase supplementation and administration route effects in vivo DNase activity, further study is needed on whether these effects are maintained in both sexes and in aged mice. Secondly, we showed that vascular density is greater in DNase‐treated mice, potentially due to microthrombi obstruction of vessels. This was shown by post‐fixation perfusion with albumin‐FITC. Unfortunately, this post‐fixation perfusion method is not a perfect representation of abnormal perfusion due to NETs after MCAO. Nevertheless, this method, by filling the entire vascular lumen, enables us to visualize changes in the vascular structure of the whole mouse brain after stroke without further invasive techniques or vessel affection. Lastly, we were pleased to reach statistical significance in spite of the small sample size of aged animals included in the analyses. Future studies with increased sample sizes are needed to reinforce our findings.

In summary, we explored direct intra‐arterial DNase administration as a new procedure in stroke therapeutics in aged mice of both sexes with AIS. The direct intra‐arterial DNase treatment significantly increased CBF, improved neurological function, reduced cerebral infarct volume, and decreased NETs in the penumbra of aged AIS mice. Our data demonstrate that direct intra‐arterial injection of DNase at the site of occlusion successfully provides an immediate and efficient improvement of CBF after recanalization in the treatment of LVOs. This study provides promising evidence in support of the clinical application of DNase treatments and IA administration.

## Disclosure

The authors have nothing to report.

## Conflicts of Interest

The authors declare no conflicts of interest.

## Supporting information


**Figure S1.** Images of brain blood flow: images of brain blood flow from all mice group at four time points: baseline, ischemia, reperfusion, and treatment.

**Figure S2.** TTC staining images (a: anterior, p: posterior)‐vehicle group: All TTC images from vehicle group.

**Figure S3.** TTC staining images (a: anterior, p: posterior)—DNase group: All TTC images from DNase group.

**Data S1.** Supporting information.

## Data Availability

The datasets used and/or analyzed during the current study are available from the corresponding author on request.
